# Partnering with Indigenous student co-researchers: improving research processes and outcomes

**DOI:** 10.3402/ijch.v74.27838

**Published:** 2015-07-27

**Authors:** Shelagh K. Genuis, Noreen Willows, Alexander First Nation, Cindy G. Jardine

**Affiliations:** 1 School of Public Health, University of Alberta, Edmonton, AB, Canada; 2 Faculty of Agricultural, Life & Environmental Sciences, University of Alberta, Edmonton, AB, Canada; 3 Morinville, AB, Canada

**Keywords:** adolescent, Canada, community-based participatory research, community researchers, First Nations, health promotion, Indians, North American, qualitative research, research, collaborative

## Abstract

**Objective:**

To examine the contribution of student co-researchers to a community-based participatory Photovoice investigation of Indigenous children's food-related lived experience. We examine co-researchers’ contributions to the research process, their role in knowledge co-generation and dissemination, and factors that fostered research partnership with the teenage co-researchers.

**Methods:**

High school students attending a First Nation community school in Canada were trained as research partners. They contributed to aspects of research design, conducted interviews with grades 3 and 4 Photovoice participants, and participated in data analysis and the development of a culturally relevant photobook. The study was initiated by the community's research committee. It is informed by critical consciousness theory and the positive youth development framework.

**Results:**

Student co-researchers incorporated culturally appropriate strategies as they interviewed participants. Co-researchers adopted conversational approaches, built rapport by articulating personal and cultural connections, and engaged in mentoring and health promotion as they interviewed participants. They made critical contributions to dissemination by developing photobook content that promoted the importance of traditional foods and the vital role of family and community in healthy eating practices. Relationships and “dialogic” space were important to building partnership with and promoting capacity development among youth co-researchers.

**Conclusions:**

Partnership between university researchers and Indigenous student co-researchers holds great promise for health promotion in communities. Co-researchers developed research and leadership skills, gained understanding of health challenges facing their community, and initiated health and cultural promotion through the project's Photobook. This investigation supports the powerful potential of student co-researchers to meaningfully contribute to research processes and to build knowledge that is relevant and credible both within and outside of their communities. Findings have implications for youth, communities and researchers.

Researchers, policymakers and community members are increasingly interested in research that co-generates knowledge and concurrently builds empowering partnerships (1). Co-generated research is particularly relevant to North American Indigenous peoples (First Nations, Inuit and Metis; and Native American Indians and Alaskan Natives) who have negative historic associations with colonialism and have experienced marginalization through traditional research processes (2). Community-based participatory research (CBPR) is an approach that acknowledges that Indigenous peoples have privileged knowledge about their own lives and should be considered for inclusion as co-researchers with university investigators. Herein, we describe the contributions that First Nations high school student co-researchers made to a CBPR study that examined the food-related lived experience of elementary schoolchildren in the community. The CBPR study was part of a university–community research programme investigating concerns about achieving family food security^1^ in a Canadian First Nation reserve community. The inter-related issues of food insecurity and unhealthy diets among North American Indigenous children are a serious public health challenge, which is associated with high rates of obesity and chronic disease (5–7). CBPR partnership with Indigenous communities is paramount to the development of interventions to improve food security (8, 9).

This exploration of student co-researchers’ contributions occurred within the context of a Photovoice investigation (10) – a powerful participatory method that enables people to record their lives and environments through photographs, facilitates discussion and reflection, and creates opportunities for personal and/or community change (11). This investigation is one of only a few Photovoice projects that positioned youth as co-researchers with university investigators (12, 13). Furthermore, to our knowledge, in only one previous Photovoice study did university researchers partner with Indigenous students as a means of facilitating authentic reflection from younger study participants (14).

Based on the United Nations’ recognition that children have the right to express their views and participate in matters affecting them (15), researchers have increasingly sought to involve children and youth in research processes. Hart's 8-step “ladder” (16), which suggests a conceptual framework that moves from adult manipulation of children to child-initiated, shared decisions with adults, has been particularly influential. Scholars assert that higher levels of participation in decision-making will increase youth self-esteem, empathy and responsibility, as well as improve community participation and the quality of response to the issue at hand (16, 17). We describe how student co-researchers moved beyond the work of research assistants completing assigned tasks: by contributing to decision-making; by partnering in data collection, analysis and dissemination; and by shaping study conclusions. As representatives of their community and in keeping with ethical requirements for research in Indigenous communities, student co-researchers were respected as research partners with unique insight into their community (18).

## Method

The CBPR food security study was conducted at Kipohtakaw Education Centre, the kindergarten to grade 12 school in Alexander First Nation (population ~1,000), a rural Cree community in Alberta, Canada. Community members predominantly participate in a cash economy to maintain economic food security; however, land-based subsistence activities such as hunting and fishing also contribute to family food security and reinforce members’ cultural identity. The community is located 18 km from a town of 6,500 and 65 km from a large city of approximately 1,000,000. Educational attainment for residents is lower than provincial averages (19); however, Alexander First Nation has the highest high school completion rate among Treaty 6 communities in Alberta (20).

**Fig. 1:**
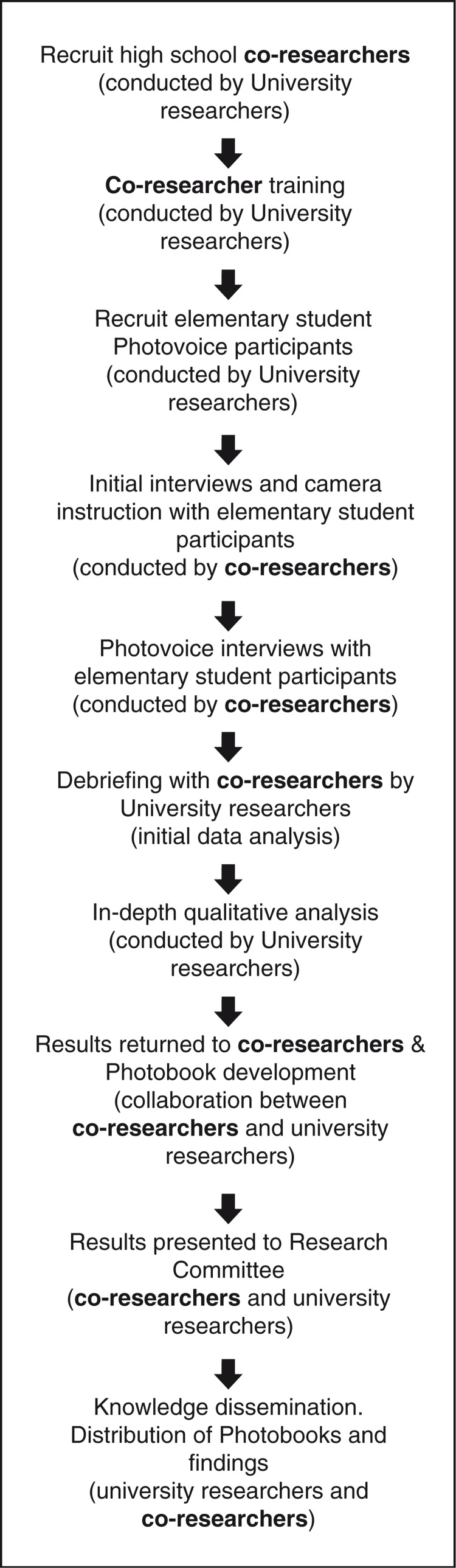
Overview of research activities.

This study focuses on the involvement and contribution of high school student co-researchers (see Fig. 1 for a summary of project activities) to a Photovoice study that sought deeper understanding of schoolchildren's food-related lived experience. The findings of the Photovoice study have been published elsewhere (10). This examination of co-researchers’ contributions is rooted in critical consciousness theory – the notion that critical examination of and reflection on life worlds will facilitate clearer understanding of the forces that shape circumstances (21). This theory emphasizes community-based identification of both problems and solutions. This study was also informed by the positive youth development framework (22), which emphasizes the potential rather than the supposed incapacities of youth and focuses on competency and skill development (23–25).

An overview of the Photovoice project is provided below as background for this current investigation. In addition to receiving ethical approval from the University of Alberta's Health Research Ethics Board, the community's research committee (consisting of community members, staff from the community's education and health departments, the school principal and university researchers) approved the research protocol. Committee members also contributed to decisions about research design, ensured appropriate cultural competency, and reviewed and approved versions of this manuscript.

### Photovoice project overview

#### Recruitment of Photovoice participants and student co-researchers

In February 2012, high school students enrolled in a “foods and nutrition” course were recruited as project co-researchers. As incentive for research involvement, students were offered credit towards classroom assignments, as well as an opportunity to positively impact their community. Nine (6 female and 3 male; aged 16–18) of a possible 14 students obtained parental consent and gave assent for research involvement. Photovoice participants were recruited in March 2012 from among the 49 students enrolled in grades 3 and 4 (aged 8–10). Parental consent and child assent were received from 33 students who were equally distributed among grades and genders.

#### Student co-researchers: training and role in the project

Student co-researchers took part in 5 training sessions with university investigators (total, 4.5 h). Training, which was carried out during class time, consisted of oral teaching, question/answer sessions, group discussion and hands-on practice. It included the following domains: research principles, Photovoice methods, interview guides, interviewing skills and recording equipment. Additionally, co-researchers were consulted about and made contributions to the interview guide (e.g. suggesting “Did you choose to eat this yourself or did someone give it to you?”) and research procedures (e.g. suggested interview settings).

To practice their interviewing skills, co-researchers conducted introductory interviews with Photovoice participants. Co-researchers recorded (in writing) information about the children's family structure and home cooking arrangements, and provided instructions about use of disposable cameras and ethical guidelines for photo-taking (e.g. “*Ask* before you take a picture of anyone”). Children were asked by co-researchers to take pictures of food they ate during an entire day, food they particularly liked or disliked and general pictures of food at home or in the community.

Student co-researchers conducted semi-structured, audio-recorded Photovoice interviews with the 26 children who returned their cameras. They asked the children to identify and discuss photos of the food they ate at the beginning of the day, during the day, for snacks, and at the end of the day. Children were asked to identify photos of things they liked to eat and things they disliked. Co-researchers asked probing questions to encourage discussion. During interviews, university researchers supported student co-researchers by occasionally helping with recording equipment and joining conversations.

Immediately following each interview session, co-researchers participated in group debriefing discussions (n=7) with university researchers. Co-researchers discussed their interviewing experiences, the interview processes and the themes they were beginning to observe. They also discussed the question: “What are you seeing or learning that you think will be important to communicate to the Research Committee and to the community?”

#### Analysis of Photovoice findings

Photovoice interviews were transcribed verbatim by a professional transcriptionist and verified by a university researcher. Data organization and analysis by university researchers was facilitated by NVivo 10™ software. Analysis incorporated conventional content analysis (26), grounded theory's constant-comparative method and examination of texts and photographs (27). The final codebook was verified by 2 university researchers and included themes identified by co-researchers during debriefing sessions, *a priori* coding guided by question categories, and inductive, open coding of interview transcripts and fieldnotes. Student co-researchers provided insight into and validated final food-related themes.

#### Photobook development and dissemination

Using at least 1 photo from each participant and a selection of participants’ and co-researchers’ words, a photobook was developed to represent Photovoice findings and facilitate research dissemination. University researchers put together the initial draft; photographs and words were arranged to proportionately represent primary themes. Over 5 lunch meetings, co-researchers made substantial changes and contributions to photobook messaging, design and content. They verified theme representation, developed health-promoting content and designed pages with culturally appropriate messages about healthy eating. The photobook was presented to the research committee by co-researchers, supported by university researchers. Photobooks have been used to animate national and international conference presentations, with co-researchers participating as co-presenters with university investigators.

### Data gathering for investigation of co-researchers’ contributions

The following data were used for this investigation of co-researchers’ activities and contributions: fieldnotes, photographs of brainstorming notes made on a white-board during training sessions with co-researchers, documentation from introductory interviews and Photovoice interview transcripts. Detailed fieldnotes, including verbatim co-researcher quotations, were recorded by one university investigator with permission throughout the project. Fieldnotes were taken on site during interactions with student co-researchers and were augmented as needed immediately following each visit to the school. Fieldnotes were reviewed by 2 university researchers.

### Analysis of co-researchers’ contributions

Themes reported in this investigation emerged through inductive open coding, conventional content analysis and grounded theory's constant comparative methods. Multiple data points (see previous paragraph) were included in the analysis to enhance rigour (28). Analysis of transcripts involved systematic examination of co-researcher/participant interactions for each interview question. The following steps were taken to strengthen credibility and facilitate transferability: triangulation between fieldnotes and transcripts; discussions with co-researchers, peers and the research committee; and careful documentation and description at all research stages. To examine the involvement and contributions of student co-researchers, we focus on their interviewing strategies, involvement in knowledge co-generation and sharing, and 2 important factors contributing to the university researcher/co-researcher partnership.

## Findings and discussion

### Enhanced interviewing strategies

During training sessions, co-researchers, all of whom were new to research and interviewing, discussed the characteristics of an effective interviewer. At the first debriefing session, co-researchers noted that interviewing was much more difficult than anticipated. They observed, for example, that the elementary students did not talk very much or spontaneously, and noted the challenge of non-verbal responses for audio-recorded interviews. Following this discussion, transcripts demonstrate that co-researchers initiated interviewing strategies that augment standard Photovoice interviewing approaches (11).

Co-researchers began to encourage participants' responses by employing rhetorical techniques that emphasized conversational, 2-way exchange. They included brief explicit affirmations and encouraged verbalization by modelling responses. Co-researchers also built rapport with the interviewees by articulating personal connections and shared community and/or cultural knowledge – for example, recognition of shared geography, built environment, and/or people. In addition, transcripts reveal that co-researchers engaged in mentoring and health promotion as they interviewed participants. This was accomplished by clarifying or providing information about healthy eating through conversational statements and questions (see Table I). Although the interviews were not positioned as opportunities for mentoring, co-researchers were cognizant of overarching study goals. This awareness and their investment in both the project and community may have led them to spontaneously share information that provided immediate, individually contextualized feedback about healthy eating.

**Table I:** Student co-researchers’ interviewing strategies

Strategy	Examples
Encouraging participant response	
Brief affirmations	“You like eating fruits in the morning too? That's cool” “Awesome. Was it good?”
Modelling responses	“I'll give you an example. ‘When I did this I think the most exciting part for me was taking pictures and then afterwards seeing them.’” “Like for me, I like carrots.”
Building rapport through connection	
Personal connection	“It's okay, I have a little sister too and she's annoying” “That's a good answer. I like monkeys. And I like them [bananas] because they're yellow and yellow is a pretty color.”
Community connection	“Did you take any pictures of people at home or people in your classroom with food? Okay, there's one of your brothers.” “I like Indian tacos^a^ because the only time I ever get to eat them is at pow-wows.^b^ We never make them at home.”
Mentoring role	
Informing through statements	“Yeah, it has some fruit; and it's also healthy because there's grains in there.” “There's beans. Beans are really good for you.”
Informing through questions	“It'd still be healthier than eating a big burger, right?” “So what makes the corn healthy? It's healthy because it doesn't contain bad substances they add in processed food?”

a An “Indian taco” is made from fried bannock (a First Nations baking powder biscuit), ground beef and cheese.

b A First Nations gathering that occurs in the summer months to celebrate culture and ceremony through traditional dance, drumming and ceremonial dress.

Although these approaches are not consistent with the question-and-answer patterns typically associated with interview-based research, a lack of response to interrogational interview styles among Indigenous peoples is noted in the literature (29, 30). Transcripts demonstrate that student co-researchers initiated culturally appropriate communication practices, which correspond to the “2-way exchange, characterized by the volunteering of information and then hinting for the response” that has been identified as appropriate for gathering personal information from Indigenous peoples (31).

### Knowledge co-generation and sharing

An important aspect of this study was the involvement of co-researchers in the development of a photobook representing the participants’ food-related lived experiences. This visual tool was planned as a means for disseminating research findings within the community. The co-researchers, however, envisioned the photobook as a means for both sharing findings *and* communicating health-promoting messages about traditional foods and the vital role of family and community in healthy eating.

#### Traditional foods

As debriefing sessions and photobook development meetings progressed, co-researchers moved beyond theme identification and, grounded by their intimate connection to the community, constructed meaning through their discussion of research findings and implications. Specifically, they observed a paucity of photos representing traditional (i.e. cultural) foods and began to explore what this might mean for the community.

Co-researchers observed: “[children] should know more about traditional foods,” and eating traditional foods is “not a part of people's everyday life but it should be.” … They thought that the most important thing would be for “parents to realize that their kids need to know that [traditional eating should be part of everyday life].” (Fieldnotes, 9 November 2012)

Having asserted that the photobook should present health-promoting messages, and encouraged by their power to influence the focus of project outcomes, co-researchers proposed and developed a photobook page (“Traditional foods: Important to our People and our Culture”) that modelled traditional eating. Co-researchers also suggested that the photobook include words in both Cree and English to promote and reinforce their traditional language. These findings demonstrate that co-researchers were actively situating healthy eating within the context of their culture, an approach to health promotion that is supported in the literature (5, 6). The co-researchers’ creative contributions were enthusiastically affirmed by the community's research committee.

#### Family and community

In-depth analysis supported the co-researchers’ identification of families and community as critical to children's food-related experiences (10). These influences were, however, represented in the transcripts rather than in photographs. The co-researchers asserted that it was important to make this finding accessible through visual representation in the photobook. One of the co-researchers, therefore, created a drawing (“Food traditions … passed down to us”) depicting a boy and his father hunting for moose. This was included in the photobook. The artist noted that the drawing represented the influence of parents and elders, and the importance of traditional practices and land-based teachings.

As the project progressed, co-researchers reflected on food security from a community and cultural perspective. They discussed their own experiences with traditional food-related activities – hunting, berry picking and preparing moose meat after a hunt (“messy and a lot of work”) – and began to explore their role in encouraging traditional eating practices. One co-researcher noted, for example, “[it is] important for kids to see older kids involved with traditional food.”

### Promoting partnership and encouraging capacity development

Two important factors contributed to building effective partnership with and encouraging capacity development among student co-researchers: the relationships that developed over the course of the study and “dialogic space.”

#### Relationships

Intergenerational dialogue supports CBPR goals of “engagement, practical relevance, knowledge generation and health promotion” (32). Intergenerational Photovoice studies have, however, commonly focused on adolescent–adult relationships. In this investigation, the interaction between high school and elementary school students fostered empowering opportunities for both groups. Photovoice participants engaged with health-promoting ideas that were promoted, not by adults, but by older students (33); and, co-researchers had the opportunity to both demonstrate leadership and learn about the children's experiences (“we learned a lot about the kids”). Moreover, co-researchers unanimously believed that their research involvement represented an important relational contribution to their community:

“We're going to be seeing them [the younger students] for our whole lives” … Co-researchers projected a sense of belonging and kinship with the younger students … a corporate sense of community with the younger students as a whole, a sense that interview interactions with the younger students represented a relational investment in the community itself, and the future community. (Fieldnotes, 8 June 2012)

Over the course of debriefing and photobook development sessions, the focus of the co-researchers moved from logistical challenges to personal sharing, critical consideration of environmental factors influencing healthy eating, and an “action” orientation (see Table II). This suggests an association between co-researchers’ increasing familiarity and developing relationship with university researchers, and their deeper engagement with the investigation. The co-researcher/researcher relationship began during training sessions where university researchers took the lead and co-researchers were positioned as learners. As the project progressed, co-researchers increasingly assumed the role of community expert, with university researchers becoming learners. Co-researcher expertise was demonstrated when they explained traditional foods and cultural practices to university researchers. It was also demonstrated by the photobook, which, on the initiation of co-researchers, became a tool for the promotion of cultural practices and healthy eating.

**Table II:** Co-researcher interaction with university investigators: from learning to leading

Sessions	Co-researcher involvement/contributions
Training sessions, sessions 1–5	Topics introduced and discussion led by university researchers e.g. introduction to and discussion of interviewing as a research method
Debriefing, sessions 1–2	University researchers/co-researchers discuss interview logistics and challenges e.g. the challenge of non-verbal responses to interview questions Co-researchers reflect on their interview experience e.g. ideas to improve interview strategies
Debriefing, sessions 3–5	Co-researchers begin to talk about their personal lives e.g. involvement in community cultural activity; extracurricular interests
Debriefing, sessions 6–7	Co-researchers begin to confidently identify emerging themes e.g. over representation of prepackaged vs. home-cooked meals in photos Co-researchers initiate and lead discussion about traditional eating e.g. explain cultural food-related practices to university researchers Co-researchers begin to explore environmental factors influencing healthy eating e.g. absence of fruit/vegetables in the community's only store^a^; “finances” Co-researchers identify messages to communicate to research committee e.g. children need to learn about and experience traditional foods in their everyday lives
Photobook development, sessions 1–5	Co-researchers initiate an “action” orientation for the photobook e.g. designed and developed photobook pages to include culturally relevant messages about healthy eating

a The closest grocery store selling fresh fruits and vegetables and a full range of market food choices on a year-round basis is approximately 18 km (11.2 miles) from the community.

#### Dialogic space

Scholars suggest that “dialogic space” between people facilitates reflection from multiple points of view, encourages collaborative ways of thinking (34, 35) and fosters creativity and collective action (34, 36). Our findings suggest that the deliberate time and space set aside for debriefing sessions was an important feature for fostering partnership with co-researchers and facilitating knowledge co-generation.

“Space” for dialogue was created through research design and serendipitously. After finding the co-researchers reluctant to gather for the first debriefing session, a university researcher began to bring home-prepared food to meetings with co-researchers. This delineated the time and place for conversation; encouraged co-researchers to sit down and engage in conversation; and prompted reflection on food heritage, the influence of family and community, and home cooking. Furthermore, in this context, co-researchers began to discuss their research-related observations as much with one another as with university researchers. This led to discussion of deeper issues, such as the relationship between eating and cultural identification. One co-researcher, for example, regularly ate traditional foods and, when younger, thought “everyone ate that way.” Another, noting that she ate traditional foods primarily at her grandmother's home, reflected, “I wish I could eat more traditionally.” Co-researchers concluded that if children do not know about or eat traditional foods, “It's almost like they don't know their own identity.” These conversations promoted consideration of typical North American versus traditional foods, and the relationships between the community and cultural practices.

## Implications

The process of having university investigators and students come together as co-researchers built individual and community capacity to address concerns about eating habits in the community's school-aged children. Findings have implications for youth, communities and researchers. As co-researchers, students developed new skills; interacted with health-promoting information in a positive peer context; and as evidenced by their discussion of environmental factors influencing healthy eating, began to explore the underlying forces that shape eating practices in their community – an important step towards critical consciousness (21). Moreover, based on their positioning as co-researchers and the positive reception to their input, student co-researchers were affirmed as experts on their own lives and community (37) and agents for positive change.

Communities also benefit from research that creates space for meaningful youth participation. Although co-researcher involvement was initially planned as a capacity building venture, these students changed and broadened the conversation (38), moving it beyond lived experience to health promotion. For example, their discussion of the food of the dominant Canadian society versus traditional foods of their own cultural group provided insight into the blend of modern and traditional influences that “comprise Aboriginal children's realities” (7). By seeing the findings through the eyes of the co-researchers, members of the research committee, who are in a position to bring about community change, gained insight into approaches for promoting healthy eating in the community's school.

Co-researchers also embodied the goals of community partnership and ownership – critical elements of ethical research in Indigenous contexts (18). This was reinforced by incorporating co-researcher participation in and author credit for oral presentations at 3 academic conferences; and by inclusion of community authorship on publications. The research committee encourages community members to be considered equal partners on research projects. Given the many health research projects in Indigenous communities and an increasing imperative for community/academic researcher partnerships, CBPR engagement may equip young people to take leadership in future research and/or health promotion endeavours. Moreover, knowledge co-generation through partnerships between Indigenous youth and scientists builds knowledge that is credible in both the community and academia (39).

This investigation supports the usefulness of the positive youth development framework for research with Indigenous communities. Although critics suggest that this framework pays insufficient attention to the complex factors that influence youth (40), the enthusiastic reception of the co-researchers’ insights by community leaders and at conferences affirmed the students’ capacity to contribute to health and cultural advocacy. The co-researchers’ contributions to this investigation illustrate their potential as valuable community assets (24).

## Limitations

Methods may pose limitations to this investigation: fieldnotes rather than recordings were used to document interactions between co-researchers and university researchers; and the study did not include a formal process for measuring capacity building. Furthermore, to foster a confidential and respectful partnership, we collected limited demographic information about co-researchers, thus potentially limiting generalizability. Although this study does not include examination of long-term impact, it contributes to a series of studies involving Indigenous youth and community wellness (41) and provides a foundation for further research.

## Conclusion

This article examines the involvement and contributions of Indigenous student co-researchers in a Photovoice project exploring food security in a Canadian First Nation community. Co-researchers facilitated interview effectiveness by incorporating culturally appropriate interview strategies, and contributing to findings, knowledge co-generation and research dissemination. As co-researchers, students developed research skills, gained insight into food security in their community, and initiated health and cultural promotion through the project's Photobook. This investigation supports the powerful potential of student co-researchers to meaningfully contribute to research processes and to build knowledge that is relevant and credible both within and outside of their communities.

## Footnotes

1 Food security exists when people have consistent “physical, social and economic access to sufficient safe and nutritious food that meets their dietary needs and food preferences for an active and healthy life” (3). Indigenous peoples experience food insecurity for many reasons, including colonialism, dispossession from traditional lands, environmental degradation, transitions from a subsistence to market economy and poverty (4).
